# Supplementary material for: Bioinspired extracellular vesicles embedded with black phosphorus for molecular recognition-guided biomineralization
Source: Nat Commun. 2019 Jun 27;10:2829. doi: 10.1038/s41467-019-10761-5 (PMC6597708; doi:10.1038/s41467-019-10761-5)
Supplement: Supplementary file 1 — Supplementary Information [file 41467_2019_10761_MOESM1_ESM.pdf]

Supplementary Information

Bioinspired Extracellular Vesicles Embedded with Black Phosphorus for Molecular Recognition-Guided Biomineralization

Wang *et al.*

1. Supplementary Figures

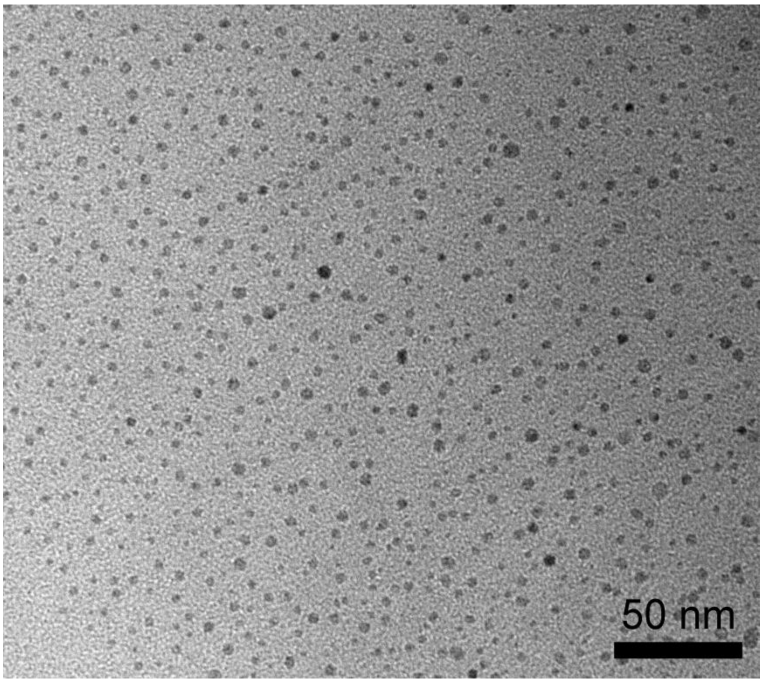

Supplementary Figure 1. TEM image of BPQDs. Source data of supplementary Figure 1 is provided as a Source Data file.

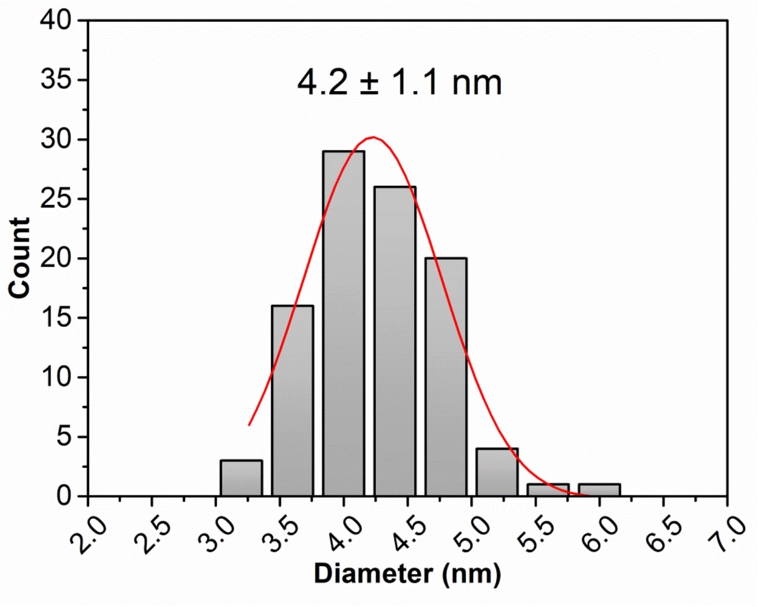

Supplementary Figure 2. Statistical analysis of the size of BPQDs based on the TEM images. Source data of supplementary Figure 2 is provided as a Source Data file.

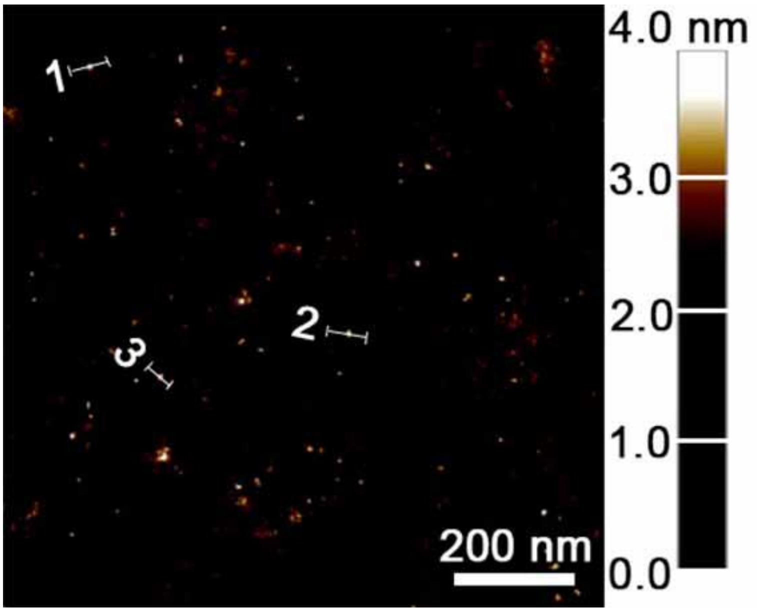

Supplementary Figure 3. AFM image of BPQDs. Source data of supplementary Figure 3 is provided as a Source Data file.

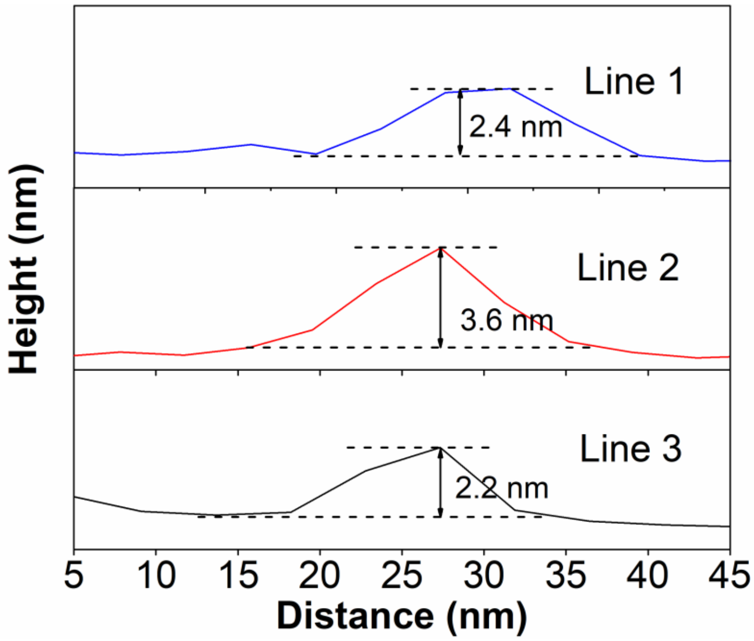

Supplementary Figure 4. Height profiles along the white lines in Supplementary Figure 3. Source data of supplementary Figure 4 is provided as a Source Data file.

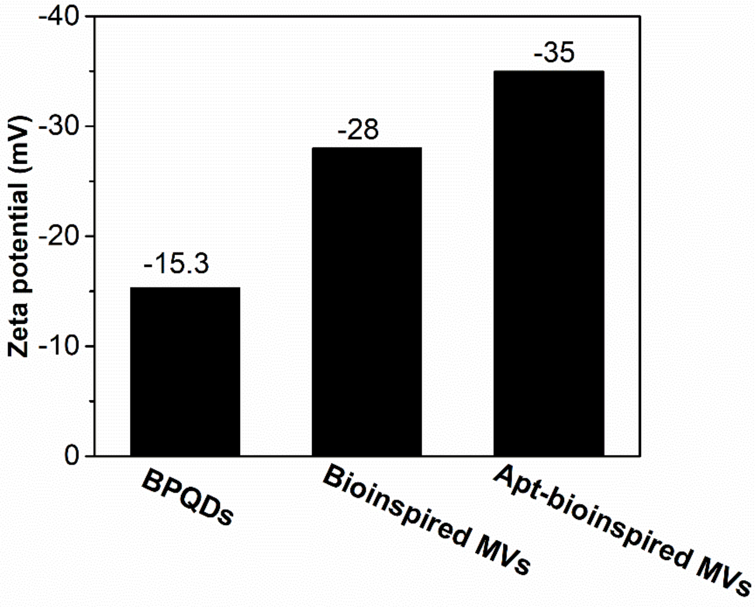

Supplementary Figure 5. Zeta potentials of BPQDs and bioinspired MVs and Apt-bioinspired MVs. Source data of supplementary Figure 5 is provided as a Source Data file.

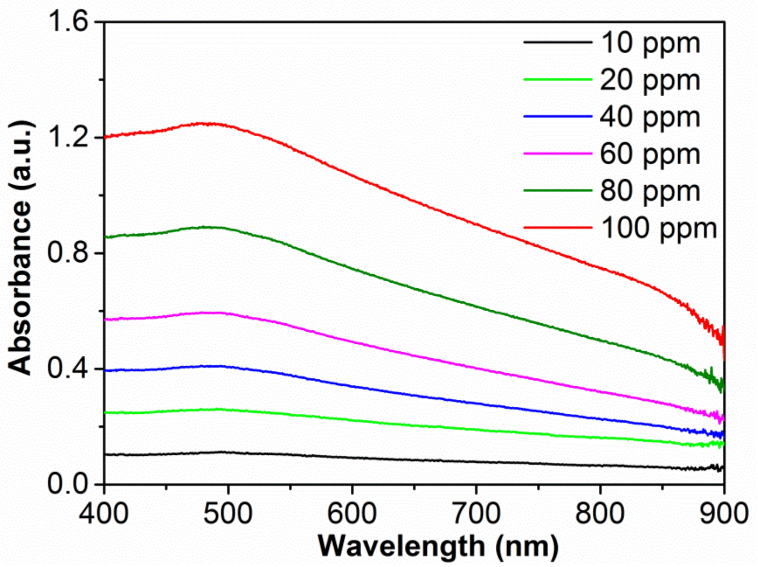

Supplementary Figure 6. UV–vis absorbance spectra of BPQDs dispersed in water at different concentrations. Source data of supplementary Figure 6 is provided as a Source Data file.

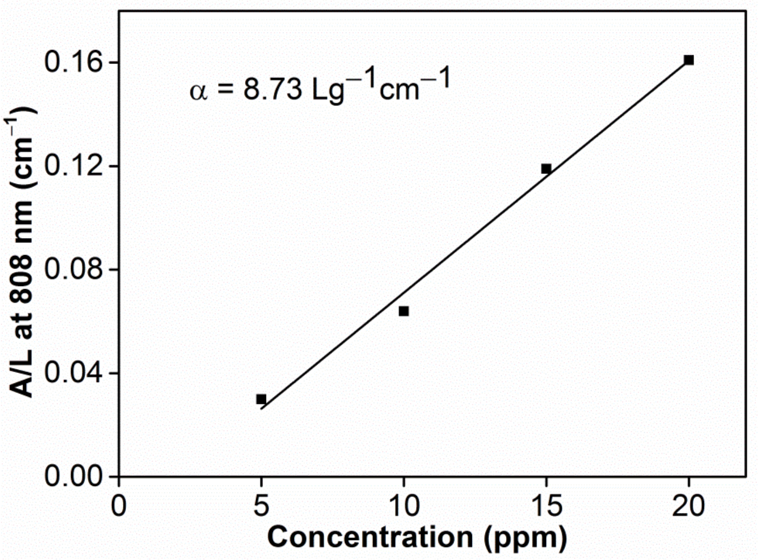

Supplementary Figure 7. Normalized absorbance intensity of BPQDs divided by the characteristic length of the cell (A/L) at different concentrations. Source data of supplementary Figure 7 is provided as a Source Data file.

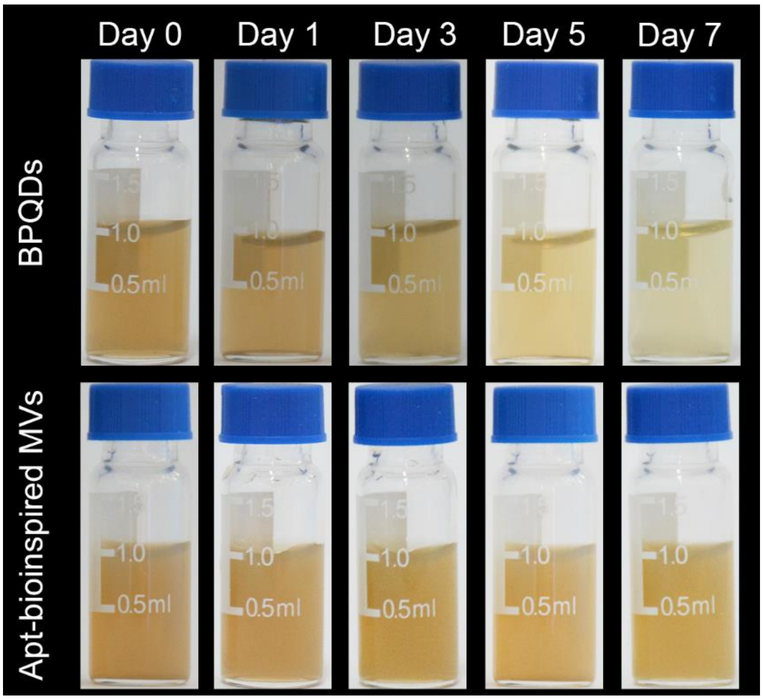

Supplementary Figure 8. Photographs of BPQDs and Apt-bioinspired MVs with the same amount of BPQDs after dispersing in water for different periods of time.

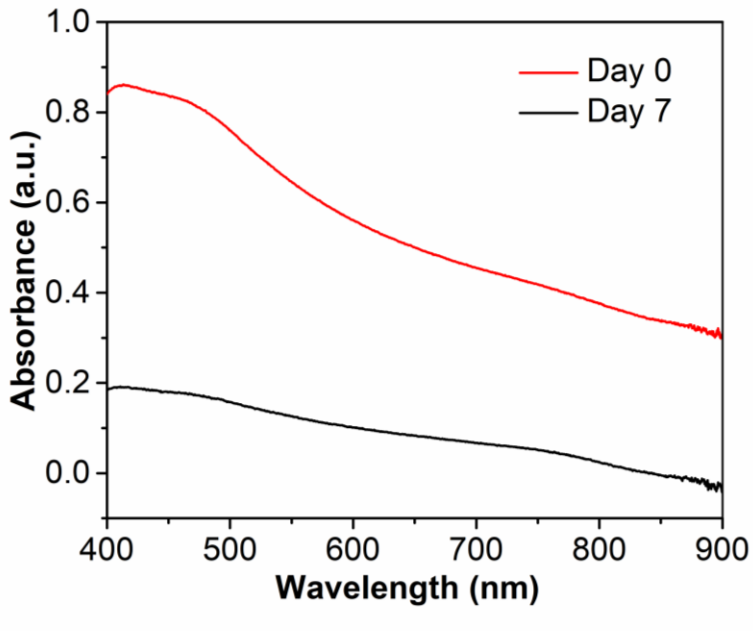

Supplementary Figure 9. Absorption spectra of BPQDs after storing in water for 0 and 7 days. Source data of supplementary Figure 9 is provided as a Source Data file.

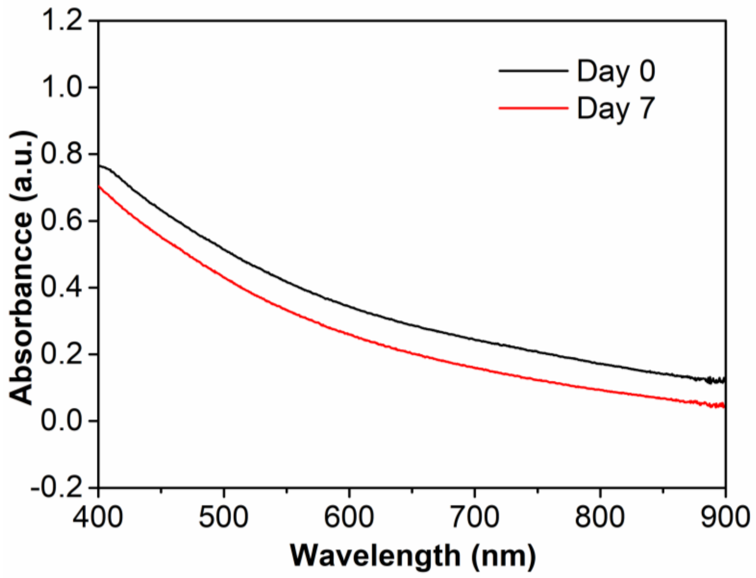

Supplementary Figure 10. Absorption spectra of Apt-bioinspired MVs after storing in water for 0 and 7 days. Source data of supplementary Figure 10 is provided as a Source Data file.

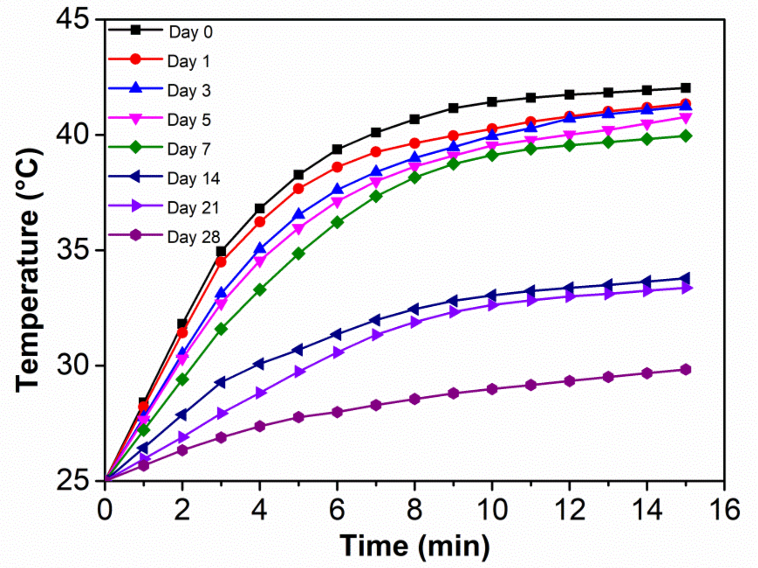

Supplementary Figure 11. Photothermal heating curves of Apt-bioinspired MVs with the 808 nm laser (1.0 W cm^{-2}) after storing in water for different periods of time. Source data of supplementary Figure 11 is provided as a Source Data file.

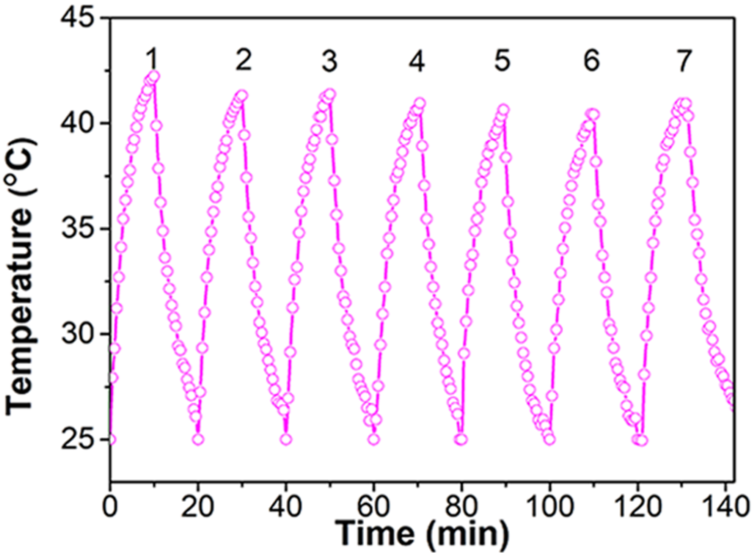

Supplementary Figure 12. Photothermal cycle curve of the Apt-bioinspired MVs dispersed in water with seven laser on/off cycles. Source data of supplementary Figure 12 is provided as a Source Data file.

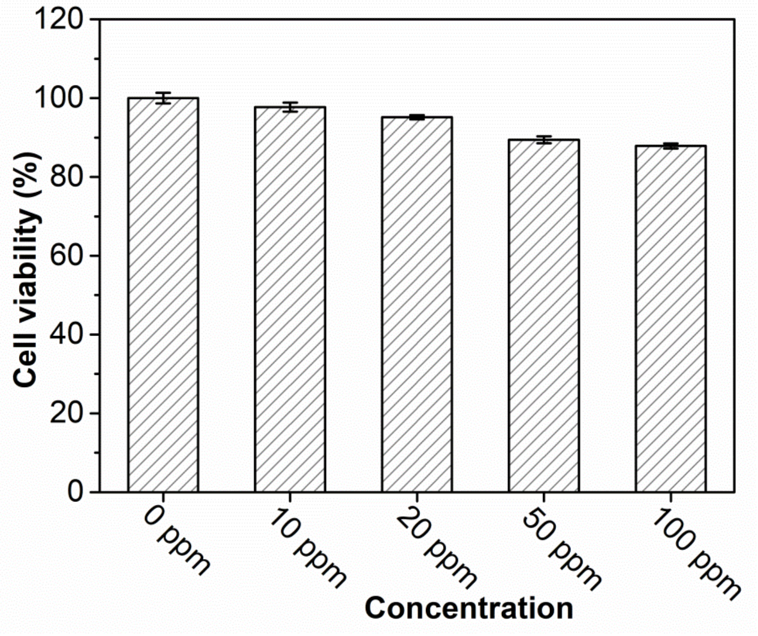

Supplementary Figure 13. Relative viability of rat osteoblasts after incubation with Apt-bioinspired MVs without NIR irradiation. Data are means ± s.d. (n=3). Source data of supplementary Figure 13 is provided as a Source Data file.

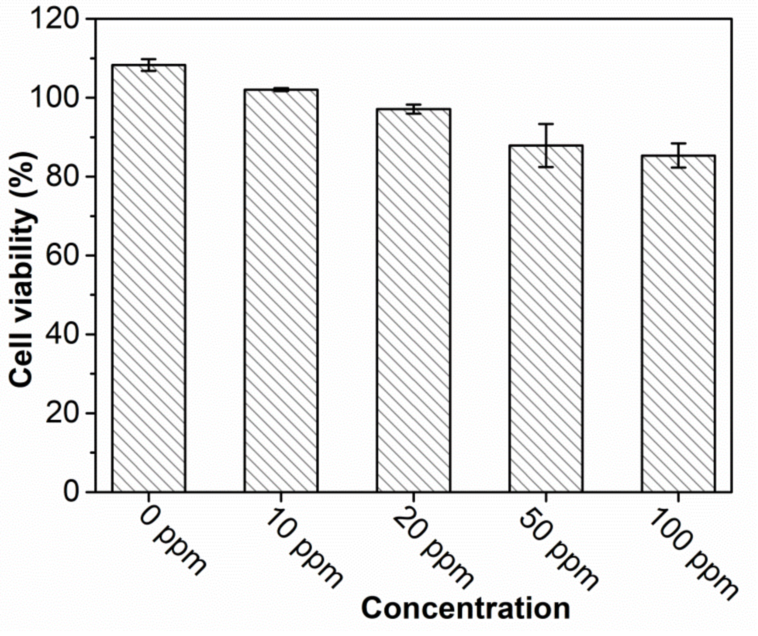

Supplementary Figure 14. Relative viability of rat osteoblasts after incubation with Apt-bioinspired MVs with NIR irradiation. Data are means ± s.d. (n=3). Source data of supplementary Figure 14 is provided as a Source Data file.

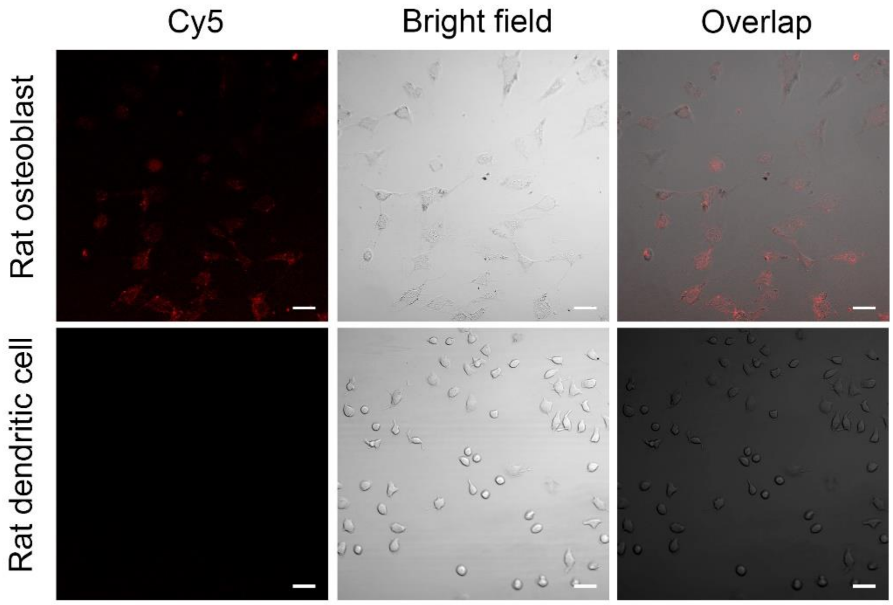

Supplementary Figure 15. Confocal microscopy images of rat osteoblasts and rat dendritic cell incubated with Cy5-labeled osteoblast-specific aptamer, respectively. Scale bars are 100 μm.

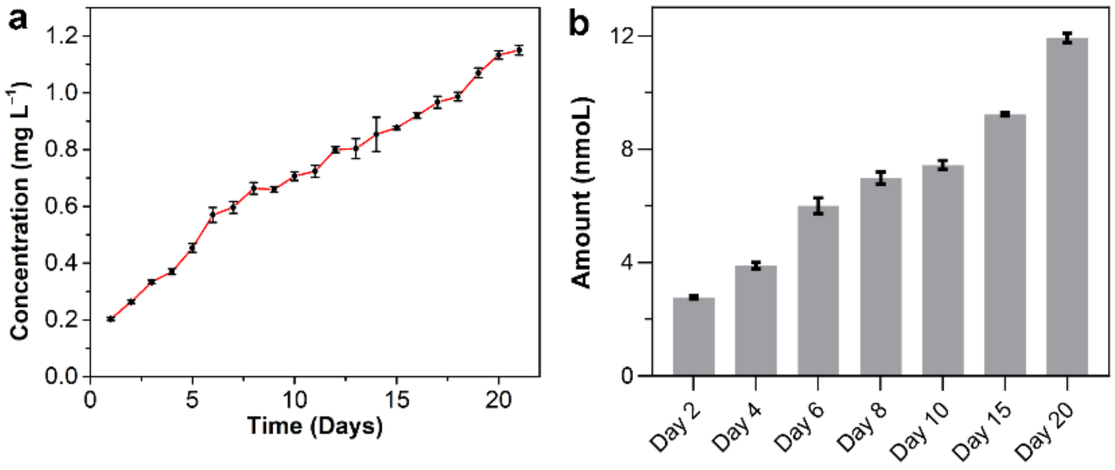

Supplementary Figure 16. (a) The concentration of phosphates released by the Apt-bioinspired MVs versus time. (b) The amount of produced phosphate ions in the in vitro degradation tests of Apt-bioinspired MVs. Data are means ± s.d. (n=3). Source data of supplementary Figure 16 is provided as a Source Data file.

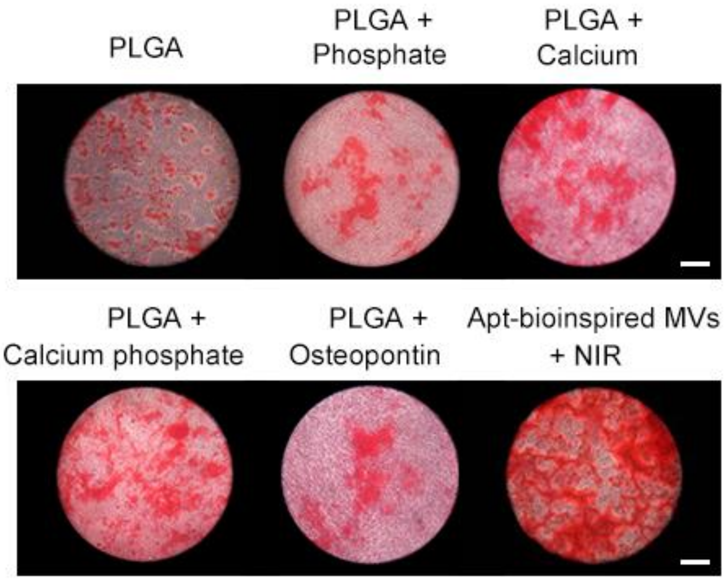

Supplementary Figure 17. Alizarin Red staining of osteoblasts treated with PLGA, osteoblasts treated with PLGA and phosphates, osteoblasts treated with PLGA and calcium ions, osteoblasts treated with PLGA and calcium phosphate, osteoblasts treated with osteopontin-embedded PLGA, and osteoblasts treated with Apt-bioinspired MVs and NIR irradiation. Scale bars are 100 μm.

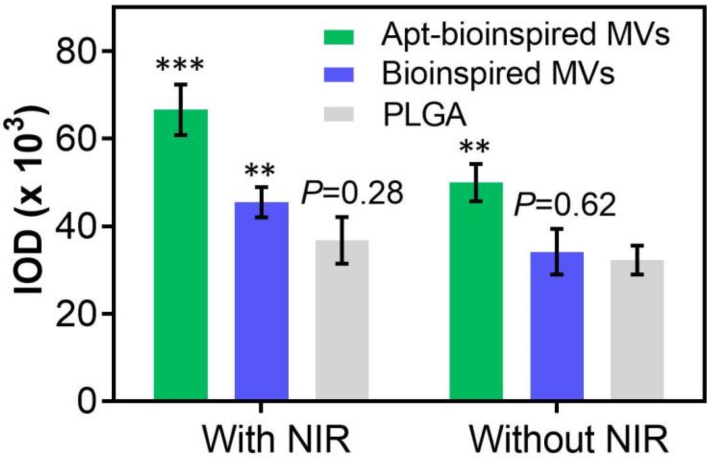

Supplementary Figure 18. ALP staining analysis of ALP expression levels in osteoblasts treated with Apt-bioinspired MVs (50 ppm), bioinspired MVs (50 ppm) and PLGA NPs (50 ppm) with or without NIR irradiation (0.2 W cm^{-2}), respectively. Data are means ± s.d. (n=3), *P<0.05, **P<0.01, *** P<0.001 (unpaired two-tailed Student's t-test). Source data of supplementary Figure 18 is provided as a Source Data file.

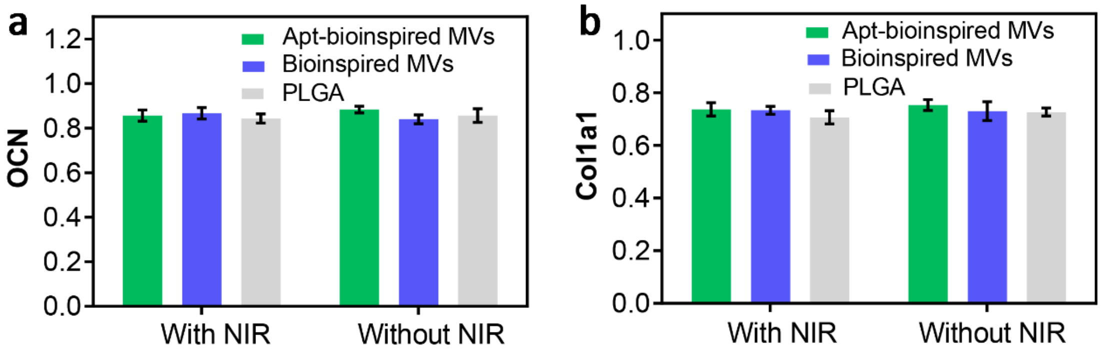

Supplementary Figure 19. (a) Western blots analysis about the ratio of OCN and (b) Col1a1 level in osteoblasts to actin in WB band images. The osteoblasts were treated with Apt-bioinspired MVs (50 ppm), bioinspired MVs (50 ppm) and PLGA NPs (50 ppm) with or without NIR irradiation (0.2 W cm^{-2}), respectively. Data are means ± s.d. (n=3). Source data of supplementary Figure 19 is provided as a Source Data file.

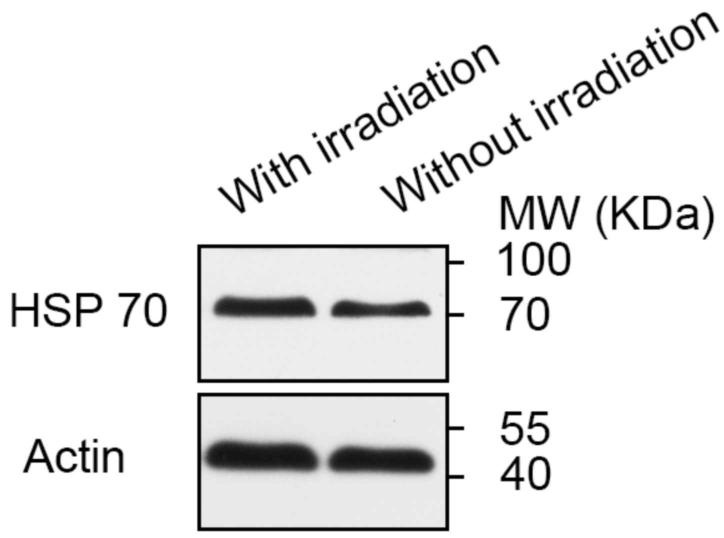

Supplementary Figure 20. Western blots analysis of HSP 70 expression levels of osteoblasts cultured with Apt-bioinspired MVs (50 ppm) with or without NIR irradiation (0.2 W cm^{-2}), respectively.

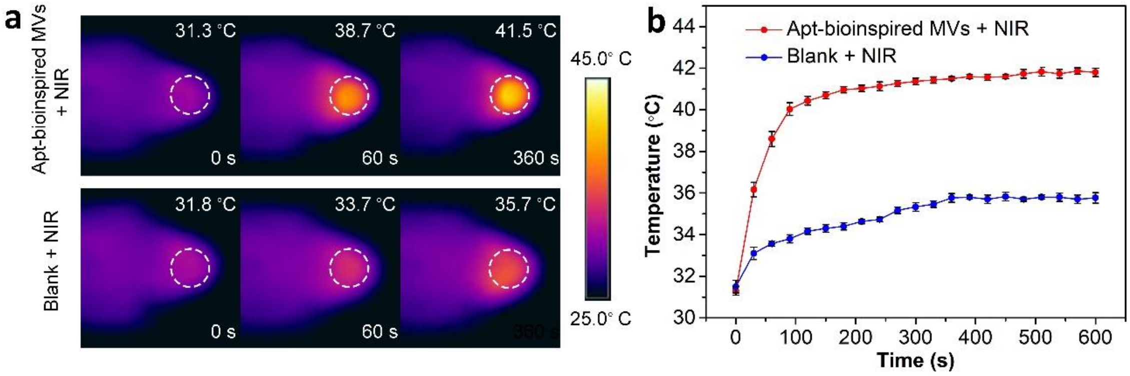

Supplementary Figure 21. (a) Infrared thermographic maps with the notations of the temperature in the NIR light (0.2 W cm^{-2} for 10 min) irradiated defect region on mice treated with Apt-bioinspired MVs and PBS, respectively. (b) Time-dependent temperature increase in the NIR light (0.2 W cm^{-2} for 10 min) irradiated defect region on mice treated with Apt-bioinspired MVs and PBS, respectively. Data are means ± s.d. (n=3). Source data of supplementary Figure 21 is provided as a Source Data file.

To investigate the molecular recognition-guided biomineralization performance of the Apt-bioinspired MVs, the Apt-bioinspired MVs were injected into mice with skull defects through the tail vein. The bone repair performance was assessed with μ-CT and H&E staining. As shown in Supplementary Fig. 22a, considerable amounts of new bone are generated in the bone defect region of mice treated with the Apt-bioinspired MVs and NIR irradiation. The H&E staining further confirms that well-arranged newly formed bone is formed in the defect region (Supplementary Fig. 22b). Compared to mice treated with Apt-bioinspired MVs and NIR irradiation, the amount of newly generated bone in the defect region is much lower in mice treated with Apt-bioinspired MVs alone (Supplementary Fig. 22c and 22d), suggesting that the hyperthermia mediated by NIR irradiation can stimulate biomineralization and bone regeneration. As for bioinspired MVs treated mice with or without NIR irradiation, only little new bone around the bone defect border is observed (Supplementary Fig. 22e-h), which indicate that the bioinspired MVs without aptamer functionalization cannot reach the bone defect region efficiently. Mice treated with NIR irradiation alone or PBS alone do not show any obvious newly generated bone, showing that the bone defect cannot be repaired by self-healing (Supplementary Fig. 22i-l). Quantitative analyses further confirms that mice treated with the Apt-bioinspired MVs and NIR irradiation shows the minimal bone defect area and the maximum bone volume fraction (Supplementary Fig. 22m and 22n).

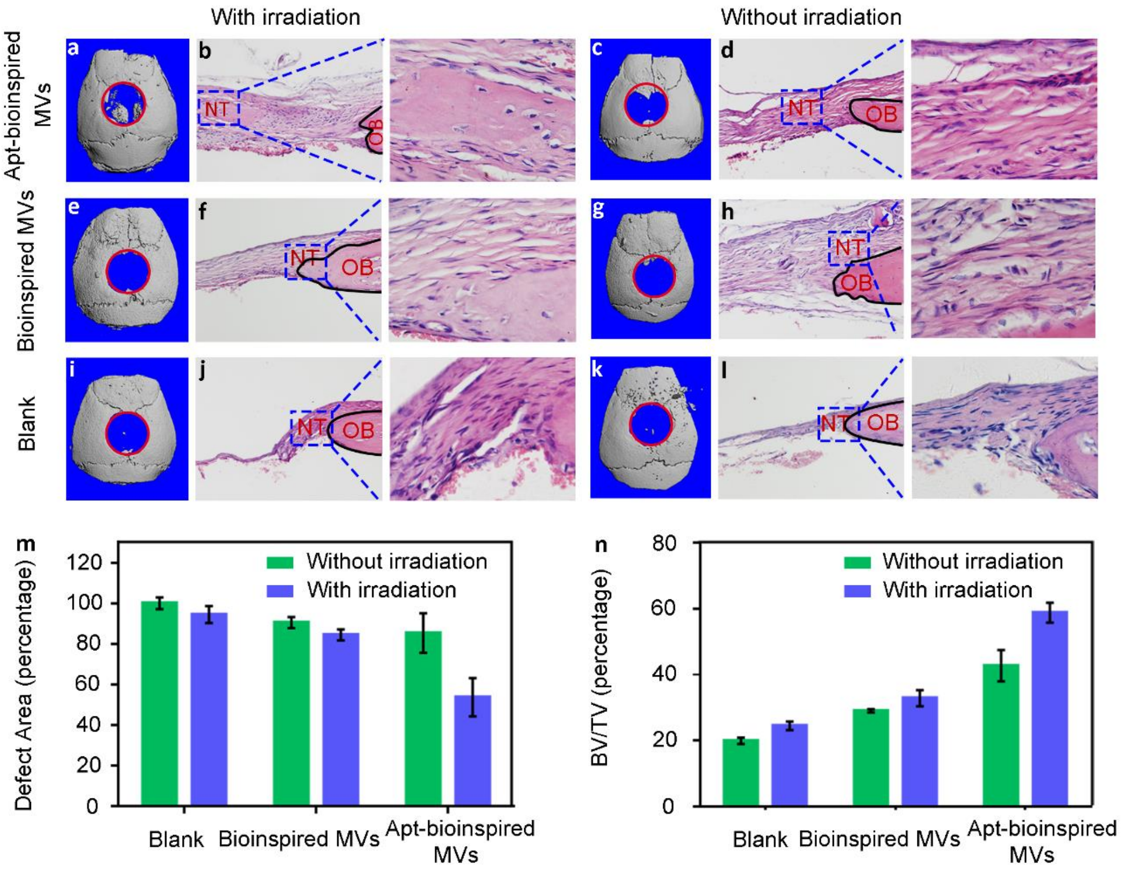

Supplementary Figure 22. ***In vivo*** **bone defect repair with intravenously injected Apt-bioinspired MVs.** (**a**) μ-CT reconstruction of the bone defect and (**b**) histomor-phological analysis of the tissue after treatment with Apt-bioinspired MVs and NIR irradiation (0.2 W cm^{-2}) for 15 min. (**c**) μ-CT reconstruction of the bone defect and (**d**) histomorphological analysis of the tissue after treatment with Apt-bioinspired MVs. (**e**) μ-CT reconstruction of the bone defect and (**f**) histomorphological analysis of the tissue after treatment with bioinspired MVs and NIR irradiation (0.2 W cm^{-2}) for 15 min. (**g**) μ-CT reconstruction of the bone defect and (**h**) histomorphological analysis of the tissue after treatment with bioinspired MVs. (**i**) μ-CT reconstruction of the bone defect and (**j**) histomorphological analysis of the tissue after treatment with nothing and NIR irradiation (0.2 W cm^{-2}) for 15 min. (**k**) μ-CT reconstruction of the bone defect and (**l**) histomorphological analysis of the tissue after treatment with nothing. The red rings represent the bone defects. The black solid lines denote the origin bone margins. NT represents newly formed tissue, and OB represents origin bone. (**m**) Quantification of defect area and (**n**) quantification of bone volume fraction (BV/TV, bone volume/tissue volume). Data are means ± s.d. (n=3), *P<0.05, **P<0.01, *** P<0.001 (unpaired t-test). Source data of supplementary Figures. 22m and 22n are provided as a Source Data file.

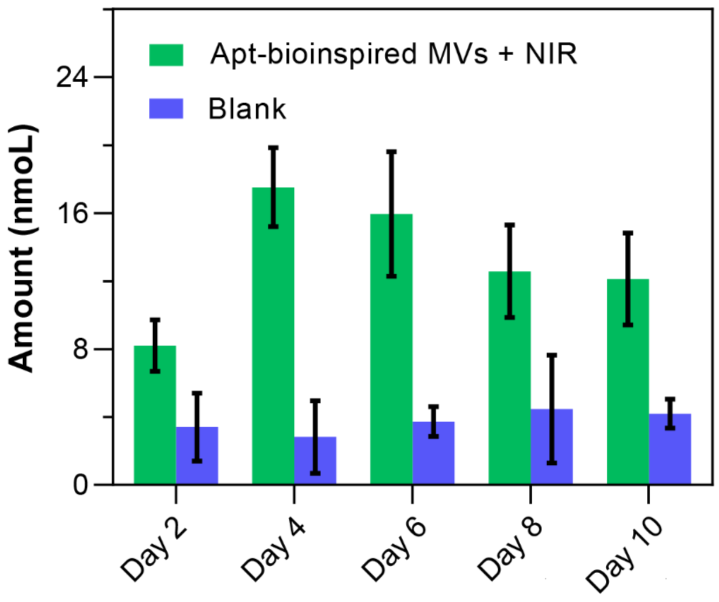

Supplementary Figure 23. The amount of produced phosphate ions in the in vivo degradation tests of Apt-bioinspired MVs. Data are means ± s.d. (n=3). Source data of supplementary Figure 23 is provided as a Source Data file.
